# Pulmonary alveolar hemorrhage mimicking a pneumopathy: a rare complication of dual antiplatelet therapy for ST elevation myocardial infarction

**DOI:** 10.11604/pamj.2016.24.308.8828

**Published:** 2016-08-11

**Authors:** Sara Oualim, Charafeddine Ait Elharda, Dounia Benzeroual, Mustapha El Hattaoui

**Affiliations:** 1 Department of Cardiology, Ibn Tofail Hospital, University Hospital Center Mohammed VI, Marrakech, Morocco

**Keywords:** Antiplatelet drugs, myocardial infarction, hemoptysis, pneumopathy

## Abstract

Diffuse alveolar hemorrhage after percutaneous coronary intervention (PCI) is a rare complication. The diagnosis is difficult and can mimic by clinical and radiological features other diagnosis as pneumopathy. We herein report the case of a 63-year-old female admitted to the hospital for ST elevation myocardial infarction. The patient underwent PCI and received dual antiplatelet therapy. Four days later, she developed dyspnea, hemoptysis and fever. Clinical, radiological and biological findings oriented to a pneumopathy and the patient received the treatment for it. Later and because of the non improvement, a thoracic computed tomography was performed and revealed patchy areas of ground-glass opacity consistent with a diffuse pulmonary hemorrhage. The combination therapy with aspirin and clopidogrel was therefore the most likely cause. Although the dual antiplatelet combination reduces systemic ischemic events after PCI, it is associated with increased risk of nonfatal and sometimes fatal bleeding. Hence the necessity of close and careful observation to watch for possible fatal complications.

## Introduction

Dual antiplatelet therapy with clopidogrel and aspirin is commonly used after percutaneous coronary intervention (PCI) in order to reduce possible systemic ischemic events and improve the outcome. This combination of antiplatelet therapy can cause bleeding complications that tend to be minor including easy bruising, petechia and ecchymosis [1]. Major bleeding events, except for gastrointestinal bleeding which is quite common and nonfatal, are very rare and might be fatal. In fact, diffuse alveolar hemorrhage is one of these rare but life-threatening major bleeding events [2]. The diagnosis can be mistaken for a pneumopathy. Herein, we report a rare case of alveolar hemorrhage caused by dual antiplatelet therapy with aspirin and clopidogrel use after PCI for ST elevation myocardial infarction mimicking a pneumopathy.

## Patient and observation

**Figure 1:**
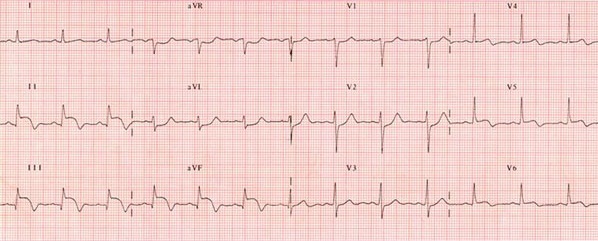
Electrocardiogram showing an ST elevation in the inferior leads

A 63-year-old female was transferred to our hospital because of chest pain. The patient had no specific pathological background. On arrival, her blood pressure was 120/75 mmHg, heart rate was 85 bpm, respiratory rate was 20/minute, and body temperature was 36.7°C. Her electrocardiogram showed an ST elevation in the inferior and basal leads (Figure 1). The patient was diagnosed as having ST elevation myocardial infarction. Transthoracic echocardiography demonstrated severe hypokinesis at the inferoseptal and posterior walls. White blood cell count was 12500/mm^3^, hemoglobin was 13.5 g/dL, and platelets were 285000/mm^3^. C- reactive protein was 25 mg/dl. Aspartate aminotransferase was 65 U/L and alanine aminotransferase was 90 U/L. Creatinine, electrolytes and hemostatis tests were all within normal limits. After the patient had taken 300 mg aspirin and 600 mg clopidogrel, she underwent primary PCI. Coronary angiograms showed 90% stenosis of the left anterior descending artery and the right coronary artery. Therefore, 2 bare metal coronary stents were implanted. The patient was admitted to the intensive care unit and dual antiplatelet therapy with aspirin at 100 mg/day and clopidogrel at 75 mg/day was started.

On the fourth day after admission, the patient coughed up bloody sputum and had a fever. The chest X-ray showed infiltrates in the upper right lobe. White blood cell count and the level of C - reactive protein were higher than the admission rates. The diagnosis of a pneumopathy was suspected and Ampicillin/ sulbactam was administrated empirically. The patient’s vital signs, symptoms, and cardiac markers improved; then the patient was transferred to cardiology general ward.

**Figure 2:**
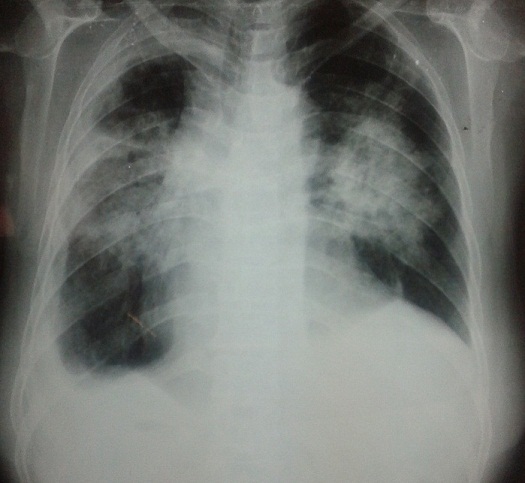
Chest X-ray revealing infiltrates in both lung fields

**Figure 3:**
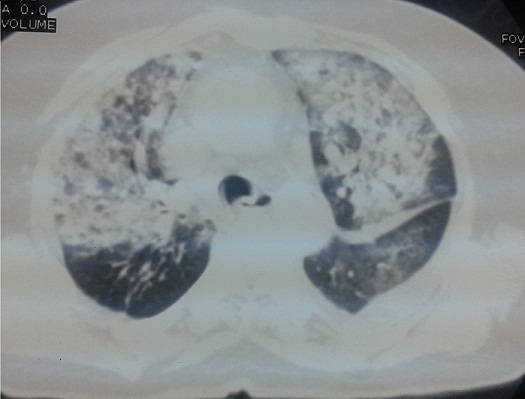
Computed tomography with enhancement showing patchy areas of ground-glass opacity in both lung fileds

On the seventh day after admission, the patient complained of dyspnea and continuous hemoptysis. The chest X-ray revealed new infiltrates in both lung fields (Figure 2). Chest computed tomography with enhancement showed patchy areas of ground-glass opacity in both lung fields (Figure 3). The findings were consistent with diffuse alveolar hemorrhage. The patient was transferred to the intensive care unit. While the dual antiplatelet therapy was suspected to be the cause of the bronchostaxis, her prothrombin time–international normalized ratio was 1.2, her platelet count and hemoglobin level remained the same and she did not need a blood transfusion. We discontinued clopidogrel and aspirin and carried out a study to discriminate between alveolar hemorrhage caused by antiplatelet therapy and alveolar hemorrhage due to a different disease. Anti-phospholipid antibody, anti cardiolipin antibody, antineutrophil cytoplasmic autoantibodies (ANCA), C-ANCA, ANA, complement 3 and 4 were all negative.

On the thirteenth day after admission, the chest radiograph showed decreased infiltration of both lung fields. We kept the patient under close surveillance. The evolution was generally good. Fifteen days later, clopidogrel was reintroduced. The patient has not developed hemorrhage signs since.

## Discussion

The syndrome of diffuse alveolar hemorrhage is characterized by bleeding into the alveolar spaces. Its etiology, pathophysiology and histological pattern allow us to categorize this syndrome into three types (pulmonary capillaritis, bland pulmonary hemorrhage and diffuse alveolar damage) [3]. Generally, the pulmonary alveolar hemorrhage is caused by Wegener’s granulomatosis, microscopic polyangitis, antiphospholipid antibody syndrome, Goodpasture’s syndrome, infection, toxin, pulmonary embolism, mitral stenosis, and malignancy. Diffuse alveolar hemorrhage induced by antiplatelet drugs is a very rare and serious complication. In the present case, the alveolar hemorrhage was at first mistaken for a pneumonia which is a common cause of pulmonary bleeding in patients with acute myocardial infarction. However, the non improvement despite the antibiotic treatment was not in favor of the pneumopathy.

In fact, alveolar hemorrhage improved only after the discontinuation of both aspirin and clopidogrel. Still, we considered the differential diagnosis of pulmonary alveolar hemorrhage due to the dual antiplatelet therapy. The patient did not have any clinical features indicating Wegener granulomatosis, microscopic polyangiitis, Goodpasture syndrome, Churg–Strauss syndrome, systemic lupus erythematosus or idiopathic pulmonary hemosiderosis, which are considered to be the most common causes of diffuse alveolar hemorrhage [3]. Besides, anti-phospholipid antibody, anti cardiolipin antibody, antineutrophil cytoplasmic autoantibodies (ANCA), C-ANCA, ANA, complement 3 and 4 were all negative. Therefore, these factors weren’t associated with the pulmonary alveolar hemorrhage in this case. Therefore, we may say that the combination of aspirin and clopidogrel was likely associated with the pulmonary alveolar hemorrhage.

The combination of dual antiplatelet decreases long-term cardiac events in patients with acute coronary syndrome undergoing PCI. According to the current treatment guidelines for ST elevation myocardial infarction, giving clopidogrel is recommended for at least 12 months. However, it is proven that the use of multiple antithrombotic drugs increases the risk of major bleeding such us pulmonary alveolar hemorrhage [4]. Because of transient hypoxemia and new radiographic infiltrates, pulmonary alveolar hemorrhage can be misdiagnosed as pneumonia or pulmonary edema. In addition, in up to one third of patients, hemoptysis may be absent at the time of presentation although it is a very important clue to a diagnosis of pulmonary alveolar hemorrhage. Fatal outcome can result of incorrect diagnosis. Early recognition and treatment of this complication is a way to improve the prognosis of the patients. To confirm the diagnosis when an alveolar hemorrhage is suspected, early bronchoscopy is a very useful tool [3], although we did not apply this option in our case.

Pulmonary alveolar hemorrhage is a very rare complication among the major bleeding events. A previous review of clinical trials in patients with acute coronary syndrome indicated that the rates of major bleeding did not significantly increase in patients receiving dual antiplatelet therapy compared to those receiving aspirin alone [5]. Although the Clopidogrel in Unstable Angina to Prevent Recurrent Events study demonstrated a significantly higher incidence of major bleeding in patients receiving the dual antiplatelet therapy, its significant reduction of cardiovascular events and its net clinical benefit favored the administration of dual antiplatelet therapy [6]. In addition, despite more widespread use of dual antiplatelet therapy and invasive cardiac procedures, a recent report showed that the rate of major bleeding has not increased over the past decade.

Pulmonary alveolar hemorrhage has been mostly reported in patients receiving glycoprotein IIb/IIIa inhibitors in association with other antiplatelet agents [7]. So far, there have only been limited reports of pulmonary alveolar hemorrhage caused solely by dual antiplatelet therapy without glycoprotein IIb/IIIa inhibitors. The first case of diffuse alveolar hemorrhage induced by clopidogrel therapy given after PCI was reported by Kilaru et al. [8]. Recently, Ikeda et al. [9] reported a case of diffuse pulmonary hemorrhage developing after combination therapy with aspirin and ticlopidine following coronary stent implantation for ST elevation myocardial infarction. In the first case, the alveolar hemorrhage was controlled by stopping the administration of clopidogrel as for our case [8], whereas pulmonary bleeding in the other case recurred and thus resulted in death [10].

## Conclusion

Dual antiplatelet therapy with aspirin and clopidogrel is a routine prescription for patients after coronary stent implantation. This case highlights that combination of antiplatelet treatment may lead to rare, life-threatening alveolar hemorrhage. The diagnosis can be mistaken with a pneumopathy. Therefore, physicians should monitor patients carefully and withdraw antithrombotic agents if an alveolar hemorrhage is suspected.

## References

[1] Roy P, Bonello L, Torguson R, de Labriolle A, Lemesle G, Slottow TL, Steinberg DH, Kaneshige K, Xue Z, Satler LF, Kent KM, Suddath WO, Pichard AD, Lindsay J, Waksman R (2008). Impact of nuisance bleeding on clopidogrel compliance in patients undergoing intracoronary drug-eluting stent implantation. Am J Cardiol..

[2] Ohkubo K, Kobayashi Y, Nakamura Y, Miyazaki A (2011). Incidence of side-effects of dual antiplatelet therapy with clopidogrel and aspirin after coronary stent implantation. Cardiovasc Intervent Ther..

[3] Ioachimescu OC, Stoller JK (2008). Diffuse alveolar hemorrhage: diagnosing it and finding the cause. Cleve Clin J Med..

[4] Eikelboom JW, Mehta SR, Anand SS, Xie C, Fox KA, Yusuf S (2006). Adverse impact of bleeding on prognosis in patients with acute coronary syndromes. Circulation.

[5] Islam AM, Patel PM (2010). Preventing serious sequelae after an acute coronary syndrome: the consequences of thrombosis versus bleeding with antiplatelet therapy. J Cardiovasc Pharmacol..

[6] Yusuf S, Zhao F, Mehta SR, Chrolavicius S, Tognoni G, Fox KK (2001). Clopidogrel in Unstable Angina to Prevent Recurrent Events Trial Investigators: effects of clopidogrel in addition to aspirin in patients with acute coronary syndromes without ST-segment elevation. N Engl J Med..

[7] Ishida R, Nomura T, Kojima A, Urakabe Y, Enomoto S, Nishikawa S, Keira N, Matsubara H, Tatsumi T (2010). Pulmonary hemorrhage in a middleaged woman as a complication of treatments for acute myocardial infarction. J Cardiol Case..

[8] Kilaru PK, Schweiger MJ, Kozman HA, Weil TR (2001). Diffuse alveolar hemorrhage after clopidogrel use. J Invasive Cardiol..

[9] Ikeda M, Tanaka H, Sadamatsu K (2011). Diffuse alveolar hemorrhage as a complication of dual antiplatelet therapy for acute coronary syndrome. Cardiovasc Revasc Med..

[10] Youngjoong Kim, Joohan Lim, Jonggu Lim, Soohyun Kim, Taeyoung Jung, Woonggil Choi (2013). Pulmonary Alveolar Hemorrhage after Clopidogrel Use for ST Elevation Myocardial Infarction. Korean Circulation Journal.

